# A rare cause of colonic pseudo-obstruction due to light chain amyloidosis

**DOI:** 10.1093/jscr/rjad517

**Published:** 2023-09-16

**Authors:** Yunpeng (Jack) Deng, Bushra Othman

**Affiliations:** Colorectal Surgical Department, Eastern Health, Box Hill, Victoria 3128, Australia; Colorectal Surgical Department, Eastern Health, Box Hill, Victoria 3128, Australia; Department of Medicine Nursing and Health Sciences, Monash University, Box Hill, Victoria 3128, Australia

## Abstract

In this case report, we discuss the rare presentation of a 56-year-old-gentleman with a history of light chain amyloidosis (AL), causing colonic pseudo-obstruction and requiring open subtotal colectomy and end ileostomy. This should remain a differential diagnosis in patients with known light chain AL presenting with nonspecific gastrointestinal symptoms such as constipation and abdominal pain. This prompts early investigation, such as endoscopy and tissue biopsy, and surgical intervention may be warranted.

## Introduction

Amyloidosis (AL) is an umbrella term used to describe a group of rare and complex diseases caused by the abnormal deposition of insoluble extracellular proteins in tissues [1]. It can affect any system in the body such as the heart, kidneys, nervous system, skin, and less commonly the gastrointestinal (GI) tract. The four most common causes of systemic AL are primary/light chain AL, Beta-2 AL, AA AL, and transthyretin-related hereditary AL [1, 2]. AL is generally a consequence of haematological malignancy, and GI involvement has a reported incidence of 3%–8% [3].

There are two main pathophysiological processes of AL which contribute to colonic stasis; alteration of the intrinsic nerve plexus, as well as damage to the mucosal vasculature, which can lead to reduced intestinal peristalsis [2, 4]. This can subsequently lead to clinical intestinal pseudo-obstruction and can even progress to an acute surgical abdomen [4].

Here, we discuss the rare presentation of a 56-year-old-gentleman found to have acute colonic pseudo-obstruction secondary to light chain AL, requiring open subtotal colectomy and end ileostomy.

## Case report

A 56-year-old gentleman from home presented with a 1-day history of fever, nonproductive cough, shortness of breath, and constipation over the last few weeks. He had a complex past medical history, including AL and multiple myeloma, being treated with immunotherapy and chemotherapy (Isatuximab, pomalidomide, and dexamethasone), as well as recurrent right pleural effusion due to AL, thalassemia minor, atrial fibrillation, and obstructive sleep apnoea.

He was initially admitted under the haematology team with consultation from infectious disease and respiratory teams for management of persistent right pleural effusion for which he required a right intercostal catheter and bronchoscopy.

**Figure 1:**
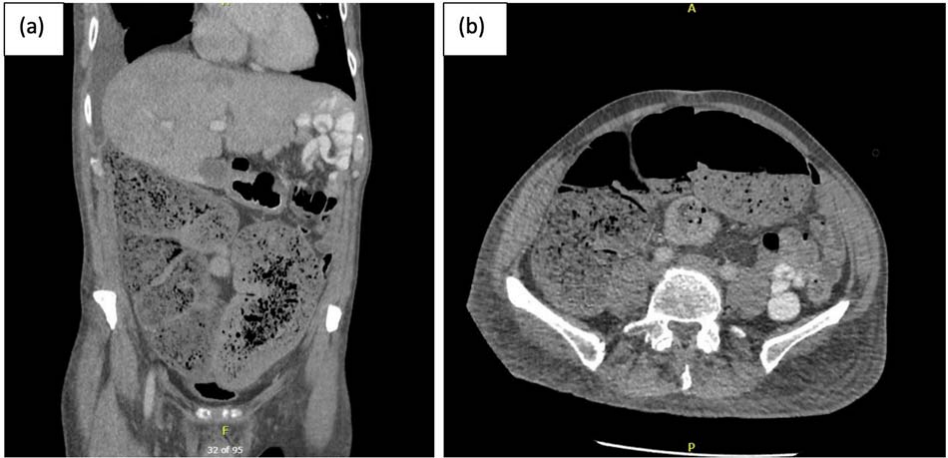
(a) Coronal and (b) axial views of CT abdominal pelvis, with oral and intravenous contrast demonstrating significant faecal loading in ascending colon and proximal to mid-transverse colon.

A week later, the patient reported new central abdominal pain with associated nausea and his last bowel action was almost 1 week prior. On examination, his abdomen was distended with generalized tenderness, but there was no peritonism. He was subsequently treated with regular oral laxatives, but with no improvement. A computed tomography (CT) abdomen and pelvis with oral and intravenous contrast was performed, which demonstrated marked faecal loading in the ascending and transverse colon with no convincing evidence of mechanical bowel obstruction (Fig. 1).

**Figure 2:**
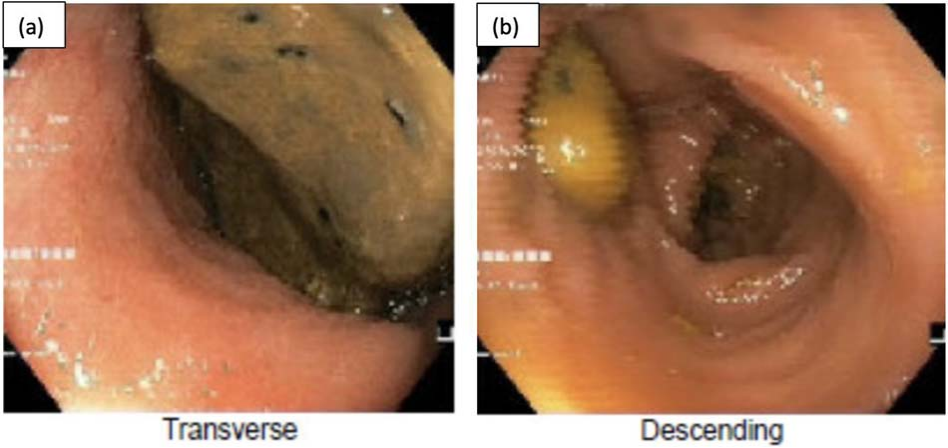
Flexible sigmoidoscopy demonstrating distended (a) transverse and (b) descending colon with faeces in the lumen and no mucosal changes to suggest ischaemia or inflammation.

The colorectal team was consulted, and the patient received increased doses of aperients and suppositories as well as regular abdominal X-ray screening to monitor for colon dilatation. Despite this, the patient had minimal clinical improvement with ongoing abdominal bloating and pain and lack of bowel action, presenting a clinical dilemma of pseudo-obstruction, or AL with colonic involvement. A decision was made to proceed with flexible sigmoidoscopy, which found distended colon with faeces in the lumen, no mucosal changes to suggest ischaemia, and no obvious mechanical obstruction (Fig. 2).

**Figure 3:**
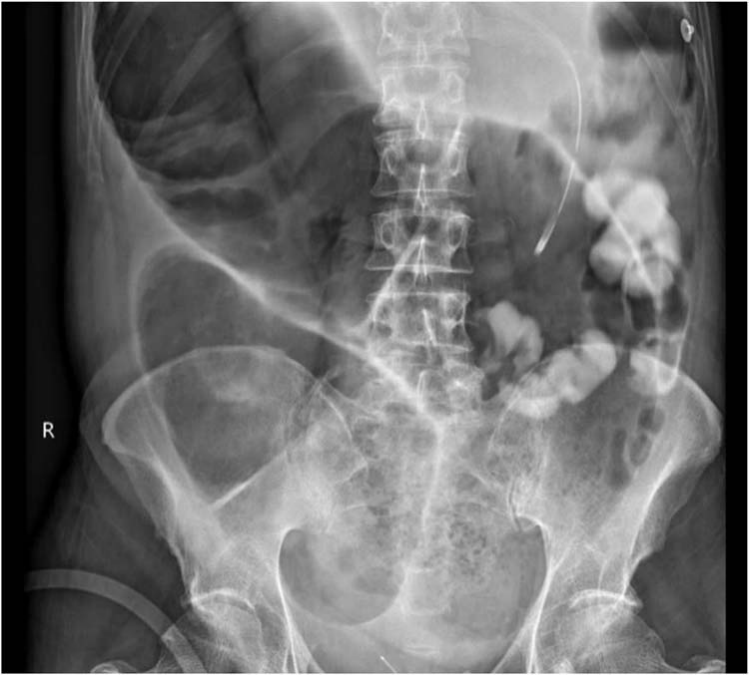
Gastrograffin study demonstrating markedly dilated gas filled loop of large bowel seen extending from the pelvis into the right upper quadrant, consistent with a dilated loop of redundant sigmoid, with no obvious contrast in the colon but some contrast in the small bowel loops in the left upper quadrant.

A subsequent gastrograffin study was performed the next day with persistent contrast in nondilated small bowel at 8 hours and nil therapeutic clinical improvement (Fig. 3).

The patient was a high-risk surgical candidate, given his comorbidities, malnutrition, and sarcopenia. He was commenced on parenteral nutrition, and a consensus decision was made to trial neostigmine to help alleviate his pseudo-obstruction. He was transferred to intensive care for close monitoring and for administration of neostigmine. Despite the several doses of neostigmine, there was no real clinical improvement, and a decision was made to proceed to surgical intervention, with the hope of performing a decompressing loop ileostomy.

**Figure 4:**
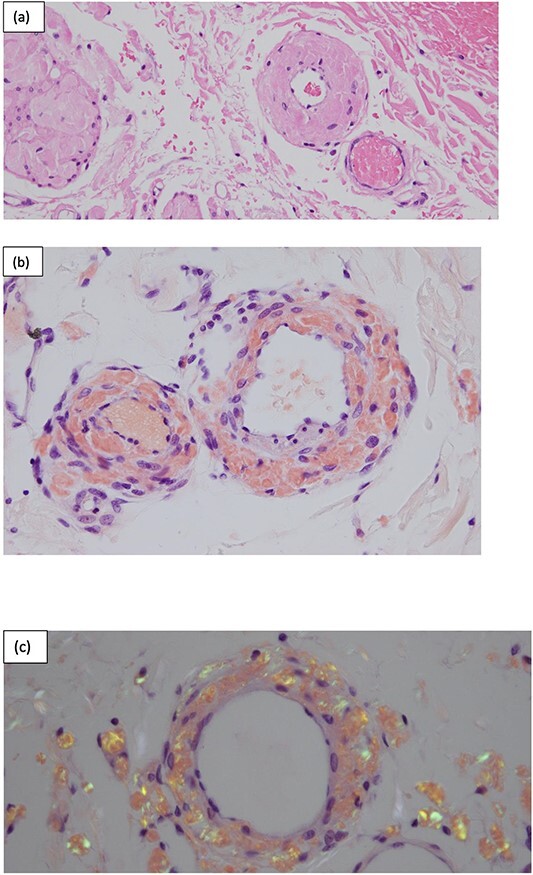
Surgical pathology of tissue. (a) Arteries within the bowel wall show marked circumferential thickening by amorphous eosinophilic material consistent with amyloid. H&E, original magnification ×200. (b) Congo red stain showing positive staining within the blood vessel wall consistent with AL. Congo red, original magnification ×200. (c) Congo red stain showing green birefringence under polarized light. Congo red with polarisation, original magnification ×200.

Intraoperatively, there was massively dilated caecum and transverse colon, with a large caecum serosal tear and multiple transverse colon serosal tears as well as a contained perforation at the hepatic flexure. The small bowel was nondilated, and there was general tapering of the left colon. A subtotal colectomy with end ileostomy was performed. Histopathology confirmed ischaemic colitis with ulceration, perforation, and peritonitis as well as a positive Congo red stain consistent with extensive AL involving blood vessels, bowel wall, and fat (Fig. 4).

Postoperatively, the patient recovered well, with initially high stoma output which settled with titration of loperamide. Patient was discharged to a rehabilitation service for ongoing optimization of physical strength and mobility.

## Discussion

Light chain AL is a systemic condition caused by haematologic malignancy, in which there is an abnormal high production of light chains produced by plasma cells in the bone marrow. It is also associated with multiple myeloma in ~15% of cases [5]. Patients with AL involving the GI tract often present with nonspecific symptoms such as nausea, altered bowel habits, and abdominal pain. Therefore, it is often a clinical challenge for doctors to make an early diagnosis.

The pathogenesis of AL and gut dysmotility is thought to be due to mucosal or neuromuscular involvement; however, this varies between different causes of AL [4, 6]. In patients with AL, amyloid deposition can occur in the muscularis mucosae, submucosa, and muscularis propria, which increases the frailty of blood vessels, thereby reducing gut wall compliance and peristalsis, which can lead to symptoms of bowel obstruction. Amyloid can also deposit in the intrinsic nerve plexus, which directly affects peristalsis and gut dysmotility [4, 6].

The gold standard for diagnosing AL requires tissue biopsy of the affected organ with Congo red staining, with a positive result confirming the diagnosis. This is typically referred to as the ‘apple green birefringence’ in polarized light, as demonstrated in Fig. 4 [2].

Therefore, in patients with suspected GI AL, endoscopy can be performed to acquire tissue biopsy to confirm the diagnosis, given there may be limited mucosal changes visible to the endoscopist. Imaging modalities to assess for systemic AL with very high sensitivity include whole-body 123I-labelled serum amyloid P scintigraphy [2]. Treatment will depend on the type and extent of AL, which can include chemotherapy or biologic therapy for definitive cure. The mainstay management of the GI symptoms remain to be supportive; however, surgical intervention may be warranted in patients with an acute surgical abdomen [2].

## Conclusion

AL with colonic involvement is a rare presentation, which can potentially progress to acute surgical abdomen, including bowel obstruction and perforation. Therefore, it should remain a differential diagnosis in patients with known AL presenting with nonspecific GI symptoms such as constipation and abdominal pain.

## Conflict of interest statement

All authors are in agreement with the contents of this submitted manuscript and that this manuscript is not being published or considered elsewhere. The authors are either in process of surgical training or have obtained FRACS in General Surgery. In addition, verbal consent was obtained from the patient and permission was gained for the use of images in this manuscript.

## Funding

The study is not funded.

## Data availability

The data underlying this article are available in the article and in its online supplementary material.
